# Novel patient engagement and recruitment strategies for an RCY of two NHS treatments for ankle osteoarthritis - total ankle replacement versus arthrodesis - the TARVA trial

**DOI:** 10.1186/1745-6215-16-S2-O94

**Published:** 2015-11-16

**Authors:** Andy Goldberg, Claire Thomson, Deirdre Brooking, Elin Rees, Marion Cumbers, Michelle Tetlow, Simon Skene, Suzie Cro

**Affiliations:** 1 UCL Institute of Orthopaedics and Musculoskeletal Sciences, London, UK; 2 Comprehensive Clinical Trials Unit at UCL, London, UK; 3 Royal National Orthopaedic Hospital NHS Trust, Middlesex, UK; 4 MRC Clinical Trials Unit at UCL, London, UK; 5 Patient & Public Representative on TARVA Trial Management Group, London, UK

## 

TARVA is an NIHR HTA funded portfolio randomised controlled trial (RCT) comparing total ankle replacement (TAR) and arthrodesis (fusion) surgery in NHS patients aged 50-85 with end-stage ankle arthritis. 328 patients will be randomly allocated to TAR or arthrodesis on an equal basis.

Clinician and patient treatment equipoise is critical to recruit surgeons and patients successfully.

The TARVA Trial team has developed novel surgeon and patient engagement techniques involving technology and multi-media tools to achieve our aims. Examples include an award-winning video, a white-labelled patient information brochure, professional newsletters and blogs, and a particular focus on the use of social networking tools to engage both investigators and patients.

The impartial trial information video featuring consultant foot and ankle surgeons and patients who have undergone TAR or fusion surgery can be accessed through the trial website (http://anklearthritis.co.uk).

The pilot phase began with the randomisation of the first patient in March 2015. An overview of our techniques alongside recruitment data accumulated until November will be presented at the ICTM conference.

