# Erratum to: ‘Novel patient engagement and recruitment strategies for an RCT of two NHS treatments for ankle osteoarthritis - total ankle replacement versus arthrodesis - the TARVA trial’

**DOI:** 10.1186/s13063-016-1218-3

**Published:** 2016-02-26

**Authors:** Andy Goldberg, Claire Thomson, Deirdre Brooking, Elin Rees, Marion Cumbers, Michelle Tetlow, Simon Skene, Suzie Cro

**Affiliations:** UCL Institute of Orthopaedics and Musculoskeletal Sciences, London, UK; Comprehensive Clinical Trials Unit at UCL, London, UK; Royal National Orthopaedic Hospital NHS Trust, Middlesex, UK; MRC Clinical Trials Unit at UCL, London, UK; Patient & Public Representative on TARVA Trial Management Group, London, UK

Unfortunately, the original version of this article [1] contained an error. The title was written as “Novel patient engagement and recruitment strategies for an RCY of two NHS treatments for ankle osteoarthritis - total ankle replacement versus arthrodesis - the TARVA trial” and should instead have been written as follows: “Novel patient engagement and recruitment strategies for an RCT of two NHS treatments for ankle osteoarthritis - total ankle replacement versus arthrodesis - the TARVA trial” where RCY should be RCT which stands for Randomised Controlled Trial.
